# Effect of physician communication on caregivers’ anxiety in pediatric trauma patients: a quasi-experimental study

**DOI:** 10.3389/fped.2026.1749878

**Published:** 2026-02-17

**Authors:** Gokhan Taskin, Ramazan Kiyak, Gozde Gunaydin Baser

**Affiliations:** Department of Emergency, Faculty of Medicine, Balikesir University, Balikesir, Türkiye

**Keywords:** anxiety, caregivers, emergency medical services, pediatric trauma, physician-patient relations

## Abstract

**Purpose:**

This study aimed to examine the effect of verbal feedback provided by the attending physician on the state anxiety level of caregivers of patients aged 0–18 years who visited the emergency department due to pediatric trauma, after the diagnosis and treatment steps became clear.

**Method:**

The sample consisted of 391 caregivers of children aged 0–18 years who visited the Emergency Department of Balıkesir University Faculty of Medicine Hospital due to trauma between July 31 and September 30, 2025. The “Personal Information Form” and the “State Anxiety Inventory” were used as data collection tools. Data were collected from caregivers of pediatric trauma patients before and after the physician briefing. Data were analyzed using a paired-samples t-test to compare pre- and post-test results. An independent samples t-test and One-way Analysis of Variance were used to examine the effect of demographic variables.

**Results:**

The mean state anxiety score of caregivers before receiving physician information was 53.31, and after receiving the information, it was 51.74. The difference between the mean scores of state anxiety was statistically significant (t = 69.876; *p* < .001). The findings indicate that explanatory feedback provided by physicians is effective in reducing anxiety in caregivers. A significant difference was found according to the mechanism of trauma; caregivers of children injured in traffic accidents had higher overall state anxiety levels compared with those whose children experienced fall- or sports-related trauma (F = 7.251; *p* < .001). However, no significant differences were found in terms of the caregivers’ degree of kinship, education level, trauma severity, and gender of children.

**Conclusions:**

The findings indicate that physician-provided verbal information is associated with a statistically significant but modest reduction in caregivers’ state anxiety levels in the pediatric emergency trauma setting. This result underscores the potential value of timely informational support for caregivers during acute care processes. This study, as one of the few empirical studies targeting caregivers’ anxiety in the field of pediatric trauma, fills an important gap in the literature and provides a strong foundation for future multicenter, longitudinal studies.

## 1 Introduction

Pediatric trauma is one of the leading causes of death and disability in childhood and accounts for a significant proportion of emergency department visits (1). Families waiting for their injured child to be treated in the emergency department may experience severe anxiety amid intense uncertainty and fear (2). The severity of the injury, uncertain prognosis, and fear of negative outcomes are the main factors that increase caregivers’ anxiety (3). This high level of caregivers’ anxiety not only affects their own psychological well-being; it can also make it difficult for them to provide accurate information to healthcare teams, participate rationally in medical decisions, and provide effective support to their children (4). Indeed, research shows that parental anxiety can directly affect the child's emotional state and care experience (5, 6). This finding indicates that supporting the family's psychological state in pediatric emergency care is critical for both the child's and the family's health.

Today, the significance of family-centered and trauma-focused approaches in pediatric trauma care has increased considerably (7). These approaches aim to actively involve families in the process and provide them with emotional support during the child's emergency treatment. For example, allowing families to be present with their children during life-threatening situations such as resuscitation, although controversial in the past, is now an increasingly common practice. Evidence suggests that family member participation in resuscitation does not negatively impact the effectiveness of medical interventions, but rather provides various benefits for both the family and the healthcare team (8). Similarly, allowing parents to be present with their children during invasive procedures in the emergency department has been shown to significantly reduce parents’ post-procedure anxiety levels and increase their satisfaction (9).

Another important aspect of family-centered trauma care is the presence of a staff member who provides families with a continuous flow of information and emotional support. Some qualitative studies have emphasized the need for a defined “parent advocate” role to guide parents through simple medical interventions to serious medical procedures such as resuscitation (7, 10). Such support helps panicked families remain calmer and regain a sense of control as they become informed about the process. The presence of a calm and supportive professional alongside the families reduces their anxiety and provides a reassuring environment for the child (11). Indeed, one of the greatest sources of anxiety in the emergency department is uncertainty and feeling uninformed. Lack of communication or inadequate information has been identified as a critical factor that increases caregivers’ anxiety (12). On the other hand, it has been found that as the amount and quality of information provided to families increases, their anxiety levels decrease. Indeed, in a randomized controlled trial of parents whose children were undergoing MRI scans under anesthesia, parents who received detailed information about the procedure and anesthesia had significantly lower State-Trait Anxiety Inventory (STAI) scores compared to controls who received only routine/minimal information (13). When communication is effective and satisfying, families feel more secure, thereby reducing their anxiety levels. In fact, regularly informing parents and involving them in the treatment process is an indispensable part of family-centered care that alleviates their worries and fears (14).

Another element of trauma-focused family care is recognizing the psychological trauma experienced by families and intervening when necessary. Families of children who have experienced severe trauma may be shaken by intense feelings of fear, helplessness, or guilt during and after the event. If these acute stress responses are not appropriately addressed, caregivers may be at risk of developing anxiety disorders or Post-Traumatic Stress Disorder (PTSD) in the future. Studies report that a significant proportion of parents of children who have experienced serious illness or injury may still exhibit trauma-related psychiatric symptoms months after the event (15). Therefore, the pediatric emergency department team must pay attention not only to the child's medical condition but also to the family's emotional state. Adopting an emergency care approach that does not ignore the psychological needs of families is a fundamental requirement that supports the healing process for both families and children.

Although family-centered initiatives are becoming increasingly common in pediatric emergency care, a recent multicenter study reported that approximately 30% of families’ emotional needs and 15% of their communication/information needs were not fully met (16). This situation highlights the need for further research to enhance the effectiveness of current practices. In summary, family support strategies that allow caregivers to be present with their child during emergency care, the presence of support staff who provide continuous information, and a sensitive approach to the family's psychological state not only reduce caregivers’ anxiety but also improve the overall quality of pediatric emergency care (7).

Despite the growing literature on family-centered care and the psychological impact of pediatric illness and trauma, limited evidence exists regarding the immediate effect of physicians’ verbal feedback on caregivers’ anxiety levels in the acute pediatric emergency setting. In particular, there is a lack of studies using a pretest–posttest design to evaluate changes in caregivers’ state anxiety following routine physician-provided information during emergency care. Therefore, this study aimed to examine the effect of verbal feedback provided by the attending physician on the state anxiety level of caregivers of patients aged 0–18 years who visited the emergency department due to pediatric trauma, after the diagnosis and treatment steps became clear. The results of this study highlight the role of verbal feedback intervention in reducing caregivers’ anxiety and the importance of establishing an evidence-based foundation for strengthening family-centered care practices in pediatric emergency departments.

## 2 Method

### 2.1 Research model

This study was designed using a single-group pretest–posttest quasi-experimental study model, one of the quantitative research methods, to examine the STAI scores of caregivers who visited the emergency department due to trauma before and after receiving a briefing from the doctor. The single-group pretest–posttest quasi-experimental study model is a research method designed to identify the relationship between different variables (17).

### 2.2 Research group

The study group consisted of caregivers of children who visited the Emergency Department of Balıkesir University Faculty of Medicine Hospital due to trauma between July 31 and September 31, 2025 (*n* = 391). Sekaran (2016) reported that working with 386 individuals in a population of *N* > 10,000 was sufficient and that the results can be generalized to the population (18). This result indicates that the results obtained from the current study sample can be generalized to similar populations. In the research, ease of access to participants was the basis for sample selection. The convenience sampling method, widely used in quantitative research, allows data to be collected quickly and at low cost (19).

### 2.3 Data collection tools

The Personal Information Form and STAI were used as data collection tools in the study.

#### 2.3.1 Personal information form

The form prepared by the researcher to identify participants’ personal information consists of seven questions: “Caregivers’ relation to Child, Age of Caregivers, Age of Child, Educational Status of Caregivers, Gender of the Child, Trauma Mechanism, and Trauma Severity.”

#### 2.3.2 State anxiety inventory

The STAI was developed by Spielberger and Gorsuch in 1964 to measure state anxiety levels in normal and abnormal individuals (20). The adaptation, validity, and reliability studies to the Turkish language were conducted by Öner and Le Compte (21). The scale consists of 20 items. The items on the scale are 4-point Likert-type items. 1 means “not at all,” while 4 means “completely.” The scores obtained from the scale theoretically range from 20 to 80. The scale items consist of direct (forward) and reverse statements. When reverse statements expressing positive emotions are scored, those with a weight of 1 are converted to 4, and those with a weight of 4 are converted to 1. In direct statements expressing negative emotions, responses with a value of 4 indicate high anxiety, while in reverse statements, responses with a value of 1 indicate low anxiety. In reverse statements, responses with a value of 4 indicate low anxiety, while responses with a value of 1 indicate high anxiety. Ten items in the State Anxiety Inventory (1, 2, 5, 8, 10, 11, 15, 16, 19, and 20) are reverse statements. The Cronbach's alpha value of the scale adapted into Turkish by Öner and Le Compte was determined to be .94 (21). In this study, the pre-test Cronbach's alpha value was found to be .91, and the post-test Cronbach's alpha value was found to be .78.

According to Table 1, the pre-test Cronbach Alpha coefficient of the STAI was found to be .91, while the post-test value was .78. Based on these results, it can be said that the STAI was a reliable measurement tool for use with caregivers of pediatric trauma patients (19).

**Table 1:** Cronbach's alpha reliability coefficient for the scale.

Scale		Number of items	Cronbach Alpha
STAI	Pre-test	20	.91
	Post-test	20	.78

### 2.4 Ethical approval

This study was ethically approved by the Balikesir University Faculty of Medicine Health Research Ethics Committee, with decision number 2025-5/2. Before the study, the participants were informed about the study's importance, and they provided informed consent.

### 2.5 Statistical analysis

In the study, data collected to determine the change in state anxiety levels of caregivers of children who visited the emergency department due to trauma before and after doctor information was analyzed using the IBM SPSS Statistics 27 program. Firstly, descriptive statistics (frequency, percentage, mean, and standard deviation) were calculated in the analysis of the data. Normality was assessed using skewness and kurtosis values together with their standardized z-scores (value/SE). Although some z-skewness and z-kurtosis values exceeded ±1.96, the skewness and kurtosis coefficients were within the ±2 range; therefore, considering the large sample size (*n* = 391) and the robustness of parametric tests to moderate deviations from normality, the data were considered approximately normally distributed and parametric statistical analyses were applied (22). To determine the change in the caregivers’ state anxiety levels, the percentage changes in the pre- and post-test mean scores were compared using a paired samples t-test. The percentage changes in participants’ anxiety levels were determined using the formula “%*Δ* = [(Pre-test–Post-test)/Pre-test]*100”. An independent samples t-test was used in the analysis of variables with two groups, and a One-Way ANOVA was used for variables with more than two groups. According to Levene's test results, the Bonferroni *post-hoc* test was used to determine the source of the difference for multiple comparisons between categorical variables with homogeneous variances. Significance was set at *p* < .05. The values found for the normality of the distributions were given in Table 2.

**Table 2:** Skewness and kurtosis values related to the normality test.

Scale		*N*	Skewness	Kurtosis
State Anxiety Inventory	Pre-test	391	−.898	.400
	Post-test	391	1,868	.857

The Skewness and Kurtosis values in Table 2 were seen to fall within the ±2 limits. It was stated that values within this range satisfy the assumption of normal distribution (22).

## 3 Results

Findings regarding caregivers’ demographic information were presented in Table 3.

**Table 3:** Descriptive statistical results regarding caregivers.

Variables	Groups	*N*	%
Relation to child	1st Degree	339	86.2
	2nd Degree	52	13.3
Education status	Primary School	16	4.1
	Middle School	27	6.9
	High School	166	42.5
	Bachelor's degree	159	40.7
	Graduate	23	5.9
Gender of children	Girl	161	41.2
	Boy	230	58.8
Trauma mechanism	Fall/Crush	160	40.9
	Traffic accident	90	23.0
	Burn	44	11.3
	Sharp/Piercing Object	40	10.2
	Sports	57	14.6
Trauma severity	Minor	267	68.3
	Moderate	113	28.9
	Major	11	2.8
Total		**391**	**100**.**0**
Age of caregivers		$\bar{X}$	**S.D.**
		38.82	11.05
Age of children		7.92	4.72

Table 4 shows that there was a significant difference between the pre-test and post-test STAI scores of Caregivers (t = 69.876; *p* < .001; Cohen's d = 3.53). The caregivers’ post-test mean scores ($\bar{X}=51.73$) were significantly lower than their pre-test mean scores ($\bar{X}=53.31$).

**Table 4:** Comparison of pre- and post-test scores on caregivers’ state anxiety levels.

Variable	*N*	$\bar{X}$	S.D.	t	*p*	Cohen's d
Pre-test	391	53.31	.44	69.876	<.001	3.53
Post-test	391	51.74	.27			

*p < .05.

According to Table 5, there was a significant difference between the percentage change levels of the caregivers’ state anxiety level pre-test and post-test averages according to the Trauma Mechanism variable (F = 7.251; *p* < .01). However, there was no significant difference in the caregivers’ relation to children (t = −.973; *p* > .05), education status (F = 2.033; *p* > .05), gender of children (t = .575; *p* > .05), and trauma severity (F = 2.705; *p* > .05). According to the trauma mechanism, the anxiety levels of those who experienced a traffic accident before and after the information session were significantly higher than those who experienced fall/collision and sports-related trauma (F = 7.251; *p* < .01). The effect size analyses revealed small effects for relationship to child, education status, gender of children, and trauma severity (Cohen's d/*η*^2^ < 0.03), whereas trauma mechanism showed a moderate effect size (*η*^2^ = 0.07).

**Table 5:** Comparison of the state anxiety levels of caregivers according to demographic variables.

Scale	Relation to child	*n*	%*Δ*		t	*p*	Cohen's d
			$\bar{X}$	S.D.			
State Anxiety Inventory Difference Score	1st degree	339	−2.96	.83	−.973	.331	.15
	2nd degree	52	−2.84	.74			
	Education status	*n*	%*Δ*		F	*p*	*η*_p_^2^
			$\bar{X}$	S.D.			
	Primary school	16	−2.56	.81	2.033	0.89	.021
	Middle school	27	−2.67	.96			
	High school	166	−2.96	.76			
	Bachelor's degree	159	−2.99	.82			
	Graduate	23	−3.10	.92			
	Gender of Children	*n*	%*Δ*		t	*p*	Cohen's d
			$\bar{X}$	S.D.			
	Girl	161	−2.92	.82	.575	.565	.06
	Boy	230	−2.97	.82			
	Trauma Mechanism	*n*	%*Δ*		F	*p*	η_p_^2^
			$\bar{X}$	S.D.			
	Fall/Crush^a^	160	−2.81	.88	7.251	.001* **b** **<** **a, e**	.07
	Traffic Accident^b^	90	−3.31	.79			
	Burn^c^	44	−2.98	.59			
	Cutting/Piercing Object^d^	40	−3.01	.63			
	Sports^e^	57	−2.71	.78			
	Trauma Severity	*n*	%*Δ*		F	*p*	η_p_^2^
			$\bar{X}$	S.D.			
	Minor	267	−2.88	.79	2.705	.068	.01
	Moderate	113	−3.07	.90			
	Major	11	−3.19	.37			

* *p* < .05; abcde: Different letters represent different groups.

## 4 Discussion

Our study found that verbal information provided by physicians to caregivers after the diagnosis and treatment process became clear in pediatric trauma cases treated in the emergency department reduced anxiety levels in caregivers. This finding is consistent with studies in the literature showing that caregivers’ anxiety is frequently observed and can be reduced through effective communication (2, 3). In a survey conducted in Canada, the average STAI level of families was found to be 38 points, which is similar to other emergency department studies (32–42 points) (16). Martin et al. reported that 42.5% of parents in Europe experienced “clinical-level” anxiety; interestingly, in the current study, the child's clinical condition or the severity of the trauma did not affect caregivers’ anxiety (2). These findings suggest that all childhood traumas create high stress in families, while the relative importance of violence may vary depending on parental perception and the quality of support provided.

The main factors triggering caregivers’ anxiety include uncertainty, lack of information, and the seriousness of the child's condition (3). In the literature, parents find the perception of the child's urgency, the prolonged recovery process, and the risk of complications most worrying (16, 23). In a 2025 study, 86% of parents accompanying their children during blood collection in the emergency department were satisfied with communication, and anxiety levels were significantly lower among parents who were informed about their child's health status (23). Similarly, other systematic reviews reported that caregivers’ anxiety increased when their children experienced conditions requiring emergency intervention, such as “poisoning, injury, or infection,” and that their stress levels rose further due to a lack of sufficient information and uncertainty about the procedures during this process (16) Since invasive procedures performed in the emergency department, such as establishing intravenous access or drawing blood, may be distressing for both children and their families, caregivers often experience increased anxiety during the evaluation process (24). In our study, caregivers’ state anxiety levels decreased significantly following physician-provided verbal information. Although the reduction in STAI scores following physicians’ verbal feedback was statistically significant, the magnitude of this change was modest, suggesting that timely informational support may help alleviate anxiety in the pediatric emergency setting.

Health literacy is an important factor determining parents’ perception of trauma and anxiety. Batool et al. reported that approximately 28.5% of parents have low health literacy and that each unit increase in literacy increases the likelihood of meeting families’ emotional needs by 9% (25). Another study reported that approximately 30% of caregivers visiting the emergency department had low health literacy (26). Low literacy levels can reduce emotional satisfaction because they make it difficult for caregivers to understand the information provided. Therefore, the literature emphasizes the importance of providing information suitable for all families using “universal measures” (such as simple language, written/graphic materials, and the restatement technique) (16). It has also been shown that parents with low health literacy tend to use the emergency department unnecessarily because they cannot understand the conditions (27). In our study, although a standardized model was not applied for physician explanations, the verbal feedback provided was associated with a significant reduction in caregivers’ state anxiety levels. This finding highlights the potential importance of verbal information during pediatric emergency care.

The current literature identifies various factors that significantly influence caregivers’ anxiety in pediatric emergency department visits. For example, it has been reported that mothers generally experience higher anxiety than fathers, and that parental anxiety scores increase significantly when the mother's educational level is low (3). Similarly, another study found that demographic variables such as the child's younger age, the mother's low educational level, the father's employment status (e.g., being unemployed), the number of children, and the family's place of residence caused significant differences in parents’ state anxiety levels (28). The quality of communication established with healthcare personnel is also seen as a determining factor in parental anxiety; indeed, it has been shown that parents who are dissatisfied with communication have higher anxiety levels, whereas anxiety can be reduced through effective communication and adequate information (29). On the other hand, the literature also contains findings suggesting that the effect of the objective severity of the trauma/illness on caregivers’ anxiety may be lower than expected (30). In fact, one study reported that the severity of the child's clinical condition did not significantly affect the family's anxiety level this suggests that caregivers’ anxiety may be more related to subjective perception and psychosocial factors.

In this study, only the trauma mechanism was found to have a significant effect on changes in anxiety levels of caregivers among the variables examined. In particular, it was observed that the difference in anxiety scores before and after the doctor's briefing was more pronounced in caregivers of children who had been in traffic accidents compared to other types of trauma. Although there is limited data in the literature on whether caregivers’ anxiety differs according to the type of trauma, it has been suggested that serious and unexpected accidents may cause more intense acute stress responses in parents. For example, it has been reported that anxiety levels are significantly elevated in parents of children who have suffered visible and significant injuries (31). On the other hand, the fact that the severity of the trauma variable was not found to be significant in this study is consistent with the aforementioned literature finding; in the study of Martin et al., the severity of the child's clinical condition did not significantly alter parental anxiety (2). Furthermore, the fact that factors such as the caregivers’ educational level, gender, and degree of relation to children did not show a significant effect on anxiety difference scores in this study does not fully correspond with the results of some previous studies. Indeed, the literature indicates that mothers are more anxious than fathers and that anxiety is higher among parents with lower educational levels (3); however, our findings did not reveal such demographic differences. This situation can be interpreted as the information and supportive communication provided by the doctor in the emergency department, resulting in a similar level of anxiety reduction in caregivers from all groups. Therefore, it can be said that accurate and timely information provides an important contribution to controlling caregivers’ anxiety, independent of factors such as the parent's educational level or whether they are a mother or father.

The effectiveness of family-centered care practices is another topic highlighted in the literature for reducing caregivers’ stress. Studies conducted in different areas, such as neonatal intensive care, have shown that family-focused interventions increase parental satisfaction while significantly reducing stress, anxiety, and depression (32). Similarly, involving families in the care process in the emergency department, providing regular information, and answering their questions meet their emotional needs. Indeed, Ali et al. reported that 15% of caregivers felt they were not sufficiently involved in the process, and 30% felt their emotional needs were not met (16). Therefore, even a simple feedback from the physician in the trauma emergency room, such as “the diagnosis has now been made, and the treatment plan is as follows,” can create significant relief for the family. In fact, in our study, we observed that physician behavior that fully explained the diagnosis and treatment steps significantly reduced caregivers’ anxiety. This practice can also prevent anxiety caused by waiting stress by informing caregivers who are experiencing difficulties with waiting times in the emergency department. A qualitative study has explained parents’ experiences and the importance of parental involvement during emergency procedures (10).

Finally, the long-term effects of pediatric trauma on caregivers’ mental health should not be overlooked. The literature reports high rates of PTSD in parents after their children experience serious injury or illness (33). For example, anxiety and depression levels in families with children who have chronic and recurrent illnesses were significantly higher than in healthy families. Balluffi et al. reported that PTSD symptoms were present in 20%–30% of parents in families with children who had burns, newly diagnosed diabetes, or a history of intensive care (34). These findings also indicate that childhood trauma can create not only short-term but also long-term psychological burdens for parents. Therefore, providing information in the emergency department not only reduces acute anxiety but can also serve as a buffer to mitigate stress responses that may arise in the future. Support and clear information provided early on can prevent parents from feeling helpless, thereby reducing the risk of chronic anxiety or PTSD developing later.

In summary, in this study, although the effectiveness of communication was not evaluated using a standardized model, physicians’ verbal feedback regarding the diagnostic and treatment process was associated with a significant reduction in caregivers’ state anxiety levels. Considering the factors that increase caregivers’ anxiety, such as uncertainty, the severity of the trauma, and health literacy issues, a family-centered approach based on effective communication is recommended in the emergency department (35, 36). Doctor-family conversations conducted as soon as the patient's condition becomes clear not only reduce immediate anxiety but also strengthen the family in the long term, protecting both the child's and the family's mental health. In this context, our study provides important support by detailing the positive impact of verbal feedback from healthcare personnel regarding the diagnostic and treatment process in pediatric trauma emergencies.

## 5 Conclusion

Our study has shown that explanatory feedback provided by physicians to caregivers of pediatric trauma patients in the emergency department significantly reduces caregivers’ state anxiety levels. This finding is consistent with the literature reporting that caregivers’ acquisition of information through effective communication with healthcare personnel reduces anxiety. The results emphasize the importance of a family-centered care approach in pediatric emergency services and demonstrate that clear, understandable information enables the process to be carried out effectively.

### 5.1 Limitations

This study has some limitations. The research was conducted at a single center and included a relatively small group of caregivers selected through convenience sampling; this may limit the generalizability of the results. Anxiety measurement was based solely on self-reported scales by caregivers; physiological stress biomarkers or observational data were not collected. Furthermore, because feedback was provided by each patient's own physician and the content and format of verbal feedback were not standardized, the potential effects of these communication differences on the results could not be controlled. The study assessed caregivers’ anxiety only in the short term (after diagnosis and treatment), and long-term effects or changes in anxiety at follow-up visits were not monitored. These limitations should be considered when interpreting the findings and applying them to larger patient groups.

### 5.2 Strengths and limitations of the study

This study is one of the few that focus on caregivers’ anxiety after pediatric trauma and address the family-centered care approach in the context of emergency services. The method used has a scientifically robust design, as it allows for the direct measurement of the effect of physician information through pre-test and post-test comparisons. The use of a highly valid scale, such as the STAI, and the fact that Cronbach's alpha values are within a reliable range for participants support the internal consistency of the data. Statistical analyses were performed using appropriate methods and demonstrated the effect of the trauma mechanism on anxiety, which is an original contribution of the study. The practical implications of the findings are also quite clear, as they show that even brief interaction with a physician in crowded clinics like emergency departments can positively affect the mental state of families.

However, the fact that the research was conducted at a single center and that participants were reached through convenience sampling limits the generalizability of the results. Since the form and content of physician information were not standardized, the communication styles of different physicians may have created variability in the results. Another weakness is that the measurement was based solely on self-report and was not supported by physiological or behavioral indicators. Furthermore, the fact that only the short-term effects of the information were examined makes it difficult to conclude the long-term course of caregivers’ anxiety. The results should be interpreted with these limitations in mind.

### 5.3 Recommendations

Future studies should be planned as multicenter investigations covering different hospitals to reach a larger and more diverse sample group. Standardizing the content, duration, and presentation format of physician information would allow for a more reliable assessment of the impact of communication. Furthermore, supporting caregivers’ anxiety not only with self-reports but also with biophysiological markers such as heart rate or cortisol levels will yield more comprehensive results. Long-term follow-up studies should be conducted to investigate the effects of this information on caregivers’ mental well-being, readmission rates, and post-traumatic stress symptoms. For families with low educational levels or limited health literacy, materials in plain language with visual support should be used, and the comprehensibility of the information should be enhanced through the use of the paralinguistic technique. Finally, providing regular training to emergency department teams on effective communication and family-centered care will ensure the sustainability of this practice and increase patient and family satisfaction.

## Data Availability

The raw data supporting the conclusions of this article will be made available by the authors, without undue reservation.
